# Improving caring quality for people with dementia in nursing homes using IPOS‐Dem: A stepped‐wedge cluster randomized controlled trial protocol

**DOI:** 10.1111/jan.14953

**Published:** 2021-07-07

**Authors:** Frank Spichiger, Andrea L. Koppitz, Susanne De Wolf‐Linder, Fliss E. M. Murtagh, Thomas Volken, Philip Larkin

**Affiliations:** ^1^ HES‐SO ▪ University of Applied Science and Arts of Western Switzerland School of Health Sciences Fribourg Fribourg Switzerland; ^2^ Faculty of Biology and Medicine Institute of Nursing UNIL University of Lausanne Lausanne Switzerland; ^3^ ZHAW Zurich University of Applied Science School of Health Professions Institute of Nursing Winterthur Switzerland; ^4^ University of Hull Hull York Medical School Wolfson Palliative Care Research Centre Hull UK; ^5^ University of Hull Hull York Medical School Hull UK; ^6^ Hull University Teaching Hospitals NHS Trust Wolfson Palliative Care Research Centre Hull UK; ^7^ ZHAW Zurich University of Applied Science School of Health Professions Institute of Health Science Winterthur Switzerland; ^8^ CHUV Lausanne University Hospital Lausanne Switzerland

**Keywords:** caring, dementia, family, needs, nursing, nursing home, palliative care, quality of life

## Abstract

**Aims:**

We aim to evaluate the effectiveness of the Integrated Palliative Care Outcome Scale for people with dementia‐based case studies to improve the caring quality for people with dementia in nursing homes by frontline staff and family members.

**Background:**

Swiss nursing homes mostly care for people with dementia. This population is at high risk of receiving little to no palliation for their complex needs. The majority of Swiss frontline healthcare staff do not systematically report on the needs of their residents. Additionally, family members do not routinely participate in assessment processes.

**Design:**

We will conduct a stepped‐wedge cluster randomized trial of repeated assessment using the Integrated Palliative Care Outcome Scale for people with dementia (IPOS‐Dem) and subsequent case studies. Clusters will consist of Swiss nursing homes randomly assigned to one of three sequential intervention time points.

**Methods:**

The study population will consist of people with dementia living in nursing homes with and without specialized dementia care facilities. Over 16 months, staff working at the frontline and family members will assess the needs and concerns of people with dementia using IPOS‐Dem. Depending on sequence allocation, facilitated case studies will start after 3, 6 or 9 months. The primary outcome will be caring quality measured by QUALIDEM. The secondary outcome will be symptoms and concerns, as indicated by the IPOS‐Dem sum‐score. The Zürich Ethics Committee approved the study in 2019 (2019‐01847).

**Impact:**

The results of this study will contribute to improving the effectiveness of person‐centred care for people with dementia. Collaboration between healthcare staff and family members will be systematically developed and built upon thorough assessment using the IPOS‐Dem and related case studies. The use of IPOS‐Dem will offer all frontline staff a systematic approach to have an independent voice within the nursing process, regardless of their qualification or grade.

## 1 INTRODUCTION

Dementia is an umbrella term for several neurological conditions that progress to impaired functioning and cognition and the inability to perform daily life activities (Livingston et al., 2020). According to Sleeman et al., (2019), people with moderate to severe stages of dementia (PWD) face the prospect of health‐related suffering. There is evidence that PWD have inadequate access to palliative care for their complex needs (Knaul et al., 2018; Sleeman et al., 2019). The complexity of caring for PWD arises from their multi‐dimensional needs, which in turn influence the individuals’ health and limit accurate prognostic assertions, palliation and treatment (Grünig, 2014, p. 9; Livingston et al., 2020). The quality of life and caring of PWD and their families are compounded by untreated and undertreated symptoms, unmet needs, and undisclosed wishes and choices (Deuschl & Maier, 2016; Husebø et al., 2011; Shim et al., 2016).

In 2009, Ecoplan (2013) estimated that about 130,000 PWD were living in Switzerland. More than 65% of PWD were aged 80 years or older. The number of PWD in Switzerland is projected to increase to 280,000 by 2050 (Ecoplan, 2013). Similarly, Livingston (2020) reported 50 million PWD worldwide, with a three‐fold increase projected by 2050. As their disease progresses, most PWD eventually move into nursing homes (Livingston et al., 2020). In Switzerland, as in most European countries, 65% of nursing home residents have dementia (Ecoplan, 2019; Onder et al., 2012).

Registered nurses (RN) in Swiss nursing homes usually provide nursing care collaborating with nursing associate professionals, healthcare assistants and other personnel (Zúñiga et al., 2021). In the German‐speaking part of Switzerland, RN make up 30% of the grade mix in nursing homes, 41% of their colleagues are nursing associate professionals, and 30% are trainees, interns and assistant staff (Zúñiga et al., 2021). Nursing homes in Switzerland have a broad organizational staff skill mix. An RN in Switzerland usually monitors the nursing process and medical management and delegates some direct care activities. Sometimes, RN in Swiss nursing homes are involved with quality and innovation (Zúñiga et al., 2021). Frontline staff in nursing homes establish caring relationships based on systematic observation of needs (Koppitz et al., 2013). For this study, and like that articulated by Watson (2008), caring is defined as a moral imperative and ethical foundation of nursing. When engaging in caring, frontline staff in nursing homes are not merely performing instrumental tasks but performing conscious and intentional forms of ‘being’ (Turkel et al., 2018; Watson, 2008).

## 2 BACKGROUND

Frontline staff in nursing homes have a challenging role; when caring for PWD, they frequently evaluate needs, medical symptoms and possibly undisclosed choices and wishes. They must monitor PWD for their needs and concerns, despite PWD having impaired cognitive abilities that influence their ability to report medical symptoms and corresponding needs (Lichtner et al., 2014). Caring in nursing homes is a collaborative task. Still, miscommunication between a staff member and a PWD or other frontline staff or family members may impede the complete detection and subsequent management of issues (Ellis‐Smith et al., 2018; Krumm et al., 2014). In Swiss nursing homes, processes for routine assessments are in place, e.g., Resident Assessment Instrument, ‘Bewohner/‐in‐ nen‐Einstufungs‐ und –Abrechnungssystem’ and ‘Planification Informatisée des Soins Infirmiers Requis’ (RAI, BESA and PLAISIR). However, the processes and policies attached to these routine assessments appear to prevent timely and relevant monitoring of PWD (Boyd et al., 2014; Koppitz et al., 2016). Two‐thirds of the frontline staff in nursing homes are not formally permitted to document their observations in routine assessments, leading to a deficit in their capacity to elicit critical concerns for PWD (Obsan, 2009; Vettori et al., 2017).

The instruments and processes in use also neglect family members’ perspectives and their consequential disclosure of current and prior decisions of PWD (Vettori et al., 2017). Processes and proposed interventions to foster family involvement in nursing homes are heterogeneous (Backhaus et al., 2020), although the unique perspectives of family members to augment the framing of quality of life, developments and caring quality of PWD are well‐documented (Ellis‐Smith et al., 2018; Robertson et al., 2019). A structured, systematic symptom and needs assessment that fosters communication between family caregivers and frontline staff may help recognize unmet needs and enable family caregivers to gain insight into PWD (Backhaus et al., 2020; Ellis‐Smith et al., 2016, 2018). The use of the Integrated Palliative care Outcome Scale for Dementia (IPOS‐Dem) helps carers to recognize symptoms and concerns of PWD (Cicely Saunders Institute (CSI), 2018a). The brief and easy‐to‐use IPOS‐Dem is built on a comprehensive family of proxy and patient‐reported outcome measures for palliative care (Cicely Saunders Institute (CSI), 2015). The IPOS‐Dem informs an overview of the quality of life and caring quality for PWD with its holistic and multi‐dimensional perspective (Ellis‐Smith et al., 2018). Quality of life for PWD is considered by disease severity and individual and environmental factors (Ettema et al., 2007b). Furthermore, Banerjee et al., (2009) conceptualized quality of life for PWD as multi‐dimensional well‐being in various domains. Therefore, the quality of life for PWD seems to be sensitive to psychological and behavioural symptoms, cognitive and functional decline and the caring quality a PWD experiences (Banerjee et al., 2009; Koppitz et al., 2013).

Ellis‐Smith (2018) reported that the use of the IPOS‐Dem improved aspects of caring quality for PWD. Quality of care, caring quality and quality of life seem closely interrelated, especially for PWD (Koppitz et al., 2013). Caring quality for PWD can be captured in the QUALIDEM.

Establishing a shared value set and management strategies based on the needs, concerns and collaborative observations of PWD is crucial to improving caring quality for PWD. Implementation of regular, systematic assessment of PWD in nursing homes is a prerequisite (Haan et al., 2018; Kupeli et al., 2016).

## 3 THE STUDY

### 3.1 Aims

We aim to evaluate the effectiveness of case studies based on a structured needs assessment using the IPOS‐Dem to improve caring quality and quality of life for PWD in Swiss‐German nursing homes by frontline staff and family members.

### 3.2 Design

We propose a stepped‐wedge cluster randomized trial (SW‐CRT) design (Hemming et al., 2015; Hemming et al., 2015) to examine the objectives. An SW‐CRT starts without exposing the clusters, nursing homes in our case, to the intervention to allow for baseline data collection. During study conduct, the clusters or cluster groups are exposed to the intervention in a step‐wise fashion, occurring after regular intervals until all clusters are exposed to the intervention. We will employ mixed‐effects models, i.e., random effects, to adjust for cluster‐specific factors and repeated measures design. This trial will evaluate whether structured symptom assessment and subsequent case studies augment caring quality for PWD. The intervention will be assessed by (1) caring quality with the QUALIDEM and (2) changes in identified needs, as assessed by the IPOS‐Dem.

The SW‐CRT has three advantages for our study. Firstly, the design allows long‐term effects, i.e., trends, to be assessed during the study (Jordan et al., 2015). One expected trend is that the caring quality, as measured by the QUALIDEM, will improve over time. The implementation of purposive data collection using the IPOS‐Dem, representing the control condition, could also be interpreted as a trend by itself, with a learning curve for frontline staff. The stepped‐wedge design will enable an objective assessment of both trends during the trial. Secondly, we aim to offer nursing home management boards added value for their residents through using an SW‐CRT because exposure to the case study intervention for 6 months is guaranteed. Thirdly, our case study intervention is dependent on the observations of frontline staff and family members using IPOS‐Dem, which promotes inclusivity and participation. Introducing the intervention en bloc was considered challenging regarding logistics regarding the aforementioned prerequisite and the complexity of the case study intervention. Therefore, we will employ measurements at fixed periods across the trial duration in participating nursing homes and consider the time effects in the models for analysis (Haines & Hemming, 2018). In other words, the presumed effect of the implementation of the IPOS‐Dem and QUALIDEM will be levelled out by the time the intervention commences.

Data collection will occur over 15 months, with frontline staff and family caregivers collecting data each month from the same participants (i.e., a closed cohort). We will randomly allocate participating nursing homes to three groups. The three groups will cross over at different time points from the control condition to the intervention condition; they will cross over with steps 3, 6 and 9 months after the trial starts. In an SW‐CRT design, the nursing homes can enrol in the study at different absolute time points as long as the planned sequence of measurement, conditions and steps are followed through as planned. An SW‐CRT, therefore, greatly improves the feasibility for the study team.

### 3.3 Study settings

Nursing homes in the German‐speaking part of Switzerland, with and without a dementia unit, will be invited to participate in the study. Participating nursing homes will be randomly assigned to one of three sequences using a random number generator from the base 3.6.2 R‐package (R Core Team, 2020).

### 3.4 Participants

Frontline nursing home staff and family members of PWD will be involved in this study. All inclusion criteria are detailed in Table 1. Potential nursing homes were identified by data‐mining lists from cantonal websites that contained accredited nursing homes and by referrals by cantonal Alzheimer associations.

**TABLE 1:** Inclusion criteria

Participant category	Inclusion criteria
People with dementia living in the nursing home	Not hospitalized during the recruitment phase and therefore physically present in the nursing home at the commencement of the study AND Diagnosis of vascular dementia or Alzheimer's disease OR People with symptoms indicating dementia which is documented in the nursing home records (BESA and RAI‐NH)
Family member of person with dementia	A family member/legal guardian of people with dementia as described above
Nursing home frontline staff	At least 18 years of age Employed at least 3 months in the respective organisation Must work at least 1 day per working week Able to communicate in German and follow the procedures of the study Provide continuing care to people with dementia
Nursing home	Provide continuing care to at least eight people with dementia

After obtaining consent from the nursing home management board, the participating nursing homes will assign a local clinical champion (Antunes et al., 2014; Bausewein et al., 2018). The clinical champion will screen subjects for eligibility using the criteria listed below and invite frontline staff, family members, attorneys (legal representatives) and PWD to separate introductory presentations and facilitate consent procedures.

### 3.5 Intervention: IPOS‐Dem case studies

This description follows the template for intervention description and replication (TIDieR) (Hoffmann et al., 2014). Tailoring, modifications and adherence will be reported according to the last three TIDieR items in conjunction with the main results.

During the intervention, IPOS‐Dem observations from frontline staff and family members will be discussed during case studies. Each case study will include a 15‐ to 30‐min group discussion about the symptoms and concerns rated with the IPOS‐Dem instrument. Systematic case studies in nursing homes led by an intervention nurse will be encouraged (Boyd et al., 2014; Koppitz et al., 2016; Neylon, 2015). The intervention aims to reinforce the IPOS‐Dem care process changes identified by Ellis‐Smith (2018): (a) facilitated communication and collaboration among staff and family, (b) facilitated internal communication, (c) facilitated communication with external healthcare professionals and (d) care planning and changes to care provision. Case studies will follow the completed IPOS‐Dem instrument structure. The IPOS‐Dem instrument structure enables a systematic approach to discuss and reflect on the concrete issues of caring for PWD, despite the nursing homes’ differing local conditions. The local clinical champion will implement onsite activities (i.e., extending invitations to family members, preparing case studies and recording changes to care plans). An intervention nurse will lead moderation and deliberation during the case studies. The intervention nurse will be an advanced practice nurse with a PhD and expertise in chronic, palliative and dementia care. Frontline staff, the local clinical champion and family members will receive training (described later) to be sufficiently prepared for the case studies. On‐duty frontline staff and available family members will be present during these group case studies at the respective nursing home. The intervention nurse will lead the case studies monthly across rotating shift patterns for 12, 9 or 6 months, depending on randomization. The presence of staff and family members, the environment, and notes and resources (e.g., separate room and flip‐chart) will be adjusted according to the local conditions and regulations in the participating nursing homes. Family members are invited to attend in groups if they wish to. But they attend only the case study for their relative living in the nursing home. The intervention fidelity and adherence will be assessed using memos recorded by the intervention nurse.

### 3.6 Training

Frontline staff and family members who have provided informed consent to participate in this study will be required to attend a mandatory introductory event 1–4 weeks before the baseline assessment. Separate 2‐h introductions will be held for frontline staff and family members. They will receive training in the study intervention and be introduced to the instruments for measuring outcomes: the IPOS‐Dem and QUALIDEM. The introductions will be conducted by members of the study team experienced in teaching and supporting staff (Pinto et al., 2018; Russell et al., 2016). A video recording of the introduction will be made available to those who cannot attend the introduction event in person. Follow‐up events will be scheduled every 3 months for frontline staff and family members to ask questions and clarify uncertainties about completing the instruments.

### 3.7 Outcomes

We will assess the study outcomes in the sequence relative to the nursing home, as illustrated in Table 2. The scales and measures to be implemented are the German versions of the QUALIDEM (Dichter et al., 2011; Ettema et al., 2007a, 2007b) and the Swiss‐German adaptation of the IPOS‐Dem. Both the QUALIDEM and IPOS‐Dem will be assessed on a cluster and an individual nursing home level.

**TABLE 2:** Sequence of outcome assessment

Outcomes	Measured at the beginning of period
Socio‐demographics	T0
BESA/RAI MDS	T0
QUALIDEM	T0, T3, T6, T9, T12 and T14
IPOS‐Dem	T0, T1, T2, T3, T4, T5, T6, T7, T8, T9, T10, T11, T12, T13 and T14

#### 3.7.1 Primary outcome: Caring quality measured with the QUALIDEM

The 18‐item QUALIDEM will be completed by nurses for each PWD in a 3‐month cycle, as shown in Table 2.

The QUALIDEM was developed to rate the quality of life, including caring quality for PWD living in residential settings by professional caregivers (Ettema et al., 2007b). For people with severe dementia, a short version with 18 items and a six‐subscale profile was validated (Dichter et al., 2016). The QUALIDEM subscales demonstrated Cronbach's alpha to be between 0.83 and 0.61 for the short version (Dichter et al., 2011) we will use. Ettema et al., (2007a) reported the original Dutch QUALIDEM inter‐rater reliability as modest, ranging from an intra‐class correlation (ICC) of 0.55 to 0.83 for the short version.

The individual subscale scores will be re‐calculated into a percentage score, as is usual for studies using the QUALIDEM (van Dam et al., ,^2019^, ^2020^; Dichter et al., 2015; Gräske et al., 2014; Oudman & Veurink, 2014). We will obtain an overall score by calculating the mean of the summed percentage scores.

We will calculate and illustrate longitudinal, total and individual subscale percentages for PWD to indicate the caring quality and quality of life trajectory.

#### 3.7.2 Secondary outcome: Symptoms and concerns measured with the IPOS‐Dem

We will report on the secondary outcome (symptoms and concerns) by calculating the total score composed of the 27 given IPOS‐Dem items assessed for the PWD by frontline staff and family members. IPOS‐Dem data will be collected at the beginning of every period, that is, monthly, as shown in Table 2, by frontline staff and family members.

The IPOS‐Dem is a questionnaire designed to be completed by carers. It contains 27 items about common and additional needs and concerns of PWD. Each item is scored on a five‐point scale ranging from 0 (no concern) to 4 (overwhelming) (Cicely Saunders Institute (CSI), 2018b).

The IPOS‐Dem was developed for the nursing home setting, where its introduction to routine care was established as feasible and acceptable (Ellis‐Smith et al., 2017, 2018). The reliability and validity of the Swiss‐German adaptation of the IPOS‐Dem will be assessed during the first 3 months of this project. We intend to detail the psychometric validation elsewhere.

Furthermore, subgroup comparisons between single‐item and subscale percentages or respective scores between the QUALIDEM and IPOS‐Dem will be tested.

### 3.8 Recruitment, consent and data collection

The project leaders will arrange meetings with the nursing home management boards to introduce the study. The suitability and availability of the nursing home for inclusion in the study will be discussed. If a nursing home management board agrees to participate in the study, a trial agreement will be signed by the head of the nursing home and the University of Applied Sciences and Arts Western Switzerland (HES‐SO), School of Health Fribourg.

We will provide all study participants, as outlined in Table 1, with appropriate information sheets and respective consent forms that describe the study and with sufficient information for the participants (and, if applicable, their attorneys) to make an informed decision about their participation. We will give the participants (and, if applicable, their attorneys) as much time as they need to make an informed decision.

The rationale for this study, which is to critically improve caring quality and quality of life for PWD in nursing homes in the future, will be emphasized to participants and communicated so that everyone can understand (Hanson et al., 2014). A research staff team member will also be present in the nursing homes regularly to offer flexibility in how PWD are informed about and consent to inclusion in the study (Hanson et al., 2014). Participants and study sites will be able to withdraw from the study procedures at any time. The informed consent will include information to confirm that the submitted anonymized data will be used until the decision to withdraw consent.

For eligible participants, the following baseline data will be captured:

- Demographics of PWD (age, gender, marital status, care dependency (Schmid, 2019), dementia type and severity).
- Demographics of family members (age, gender, marital status and relation to the person with dementia).
- Demographics of frontline staff (age, gender, professional role, highest completed education, type of palliative care training and the number of years of working experience).
- The latest documentation within RAI or BESA.

The clinical champion assigned in each nursing home (Antunes et al., 2014; Bausewein et al., 2018) will primarily oversee the measurements of the IPOS‐Dem and QUALIDEM. The IPOS‐Dem measurement will take place monthly (T0–T14). A study team member will be responsible for ensuring that all the scheduled measurements are conducted by reminding and supporting the clinical champions and the clinical contact person at each site. The clinical champion will ensure that the QUALIDEM is measured and completed at the time points specified in Table 2.

### 3.9 Sample size calculation

The sample size was calculated using simulations for a cohort stepped‐wedge design. The primary outcome would be repeatedly obtained from the same participants within each cluster (nursing home), as in previous similar studies (Aegerter et al., 2020). Based on previous QUALIDEM results, we assumed a baseline mean of 71.3 and a standard deviation of 16.7 (van Dam et al., 2019). The number of nursing homes was set to 20 with three cluster groups. The attributable difference in the QUALIDEM was expected to be around 5%, as derived from clinical expertise. We set the number of steps to three for practical reasons (study rollout). After baseline measurement, nursing homes will switch from the control condition to the intervention condition at given periods until all nursing homes are applying the intervention regime. To determine the appropriate sample size, we varied the average cluster size in our simulation over a range of 4 to 16 subjects per cluster. We assessed the sensitivity of the sample size calculations by varying the cluster‐specific and subject‐specific ICC. More specifically, we used ICC values for Rho[1] = {0.1, 0.2} and Rho[2] = {0.2, 0.3}, respectively. In our simulation, we used a standard closed cohort mixed‐effects model. The latter comprised a random effect for the clusters (*α*) and a random effect for the repeated measurements on the same cohort of individuals (*ζ*). Moreover, the model included a fixed effect to account for time trends (*β*) and a fixed effect representing the treatment effect (*θ*).

(1) $$y=\mu_{\mathrm{ij}}+e_{\mathrm{ijk}}$$

(2) $$\mu_{\mathrm{ij}}=\mu +\alpha_{i}+\zeta_{\mathrm{ik}}+\beta_{j}+X_{\mathrm{ij}}\theta$$

(3) $$\zeta_{\mathrm{ik}}∼N\left(0,\sigma_{\zeta }^{2}\right)$$

(4) $$\alpha_{i}∼N\left(0,\sigma_{\alpha }^{2}\right)$$

(5) $$e_{\mathrm{ijk}}∼N\left(0,\sigma_{e}^{2}\right)$$

To estimate our models, we used the linear mixed‐effect method (lmer) from the R‐package lme4 (Bolker et al., 2021). Furthermore, we set the acceptance probability for a Type I Error to *p* = .05 and the acceptance probability for a Type II Error to *p* = .20 (power = 0.80).

**FIGURE 1:**
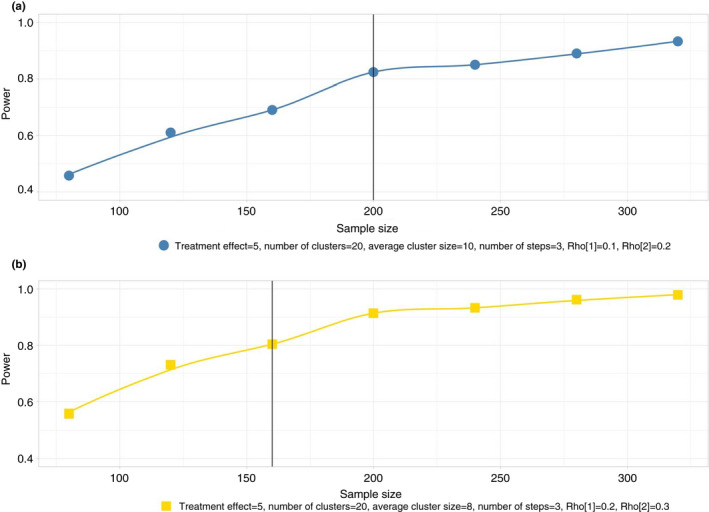
Power and sample size over a varying range of the mean number of subjects per cluster

The required sample sizes to achieve the desired power of 0.80, given the aforementioned parameters and assumptions, are shown in the two scenarios plotted in Figure 1.

Overall, a higher ICC yielded a lower total number of subjects needed to achieve the desired power (Figure 1, panel b compared with a). We found the solution with 200 participants, 20 nursing homes and three steps (Figure 1a) to be optimal because three steps put much less burden on participants and study staff than a greater number of steps. On the other hand, the proposed ICCs are more conservative. Furthermore, it seems highly feasible to administer the intervention to participants in the randomized clusters within three steps. To account for attrition, we further adjusted the required sample size. Husebø et al., (2019) and Chenoweth et al., (2011) reported 20–25% attrition. We increased the number of clusters from 20 to 22 to address the risk of underpowering our study. To summarize, our study will enrol and follow 220 participants in 22 clusters over six discrete measurements (one at baseline and five during the control and intervention conditions), which will yield a total of 1320 observations. We do not intend to conduct interim analyses of the outcomes.

### 3.10 Validity and reliability

The allocation of a nursing home to a group will be concealed from the researchers and nursing home management during the recruitment phase. Depending on local schedules for staff rotation and planning, the cross‐over date (from control to intervention) will be communicated to the intervention nurse and nursing home to allow for scheduling case studies during the intervention. Blinding participants to the intervention will not be possible due to the intervention nurse visits and scheduling of the case studies.

Introductory sessions for all nurses and family members in all nursing homes will be conducted by the same members of the study team and the intervention nurse to avoid any teaching bias. A clinical champion will be allocated to ensure data quality in terms of completeness of data and completion pattern (Bausewein et al., 2018).

The QUALIDEM and IPOS‐Dem questionnaire responses will be anonymized and entered into an electronic case reporting form (eCRF) using REDCap electronic data capture tools hosted at HES‐SO, School of Health Valais, Switzerland (Harris et al., 2009, 2019). The original papers will remain in the nursing home.

The eCRF will consist of forms and data entry fields that allow capturing of single events and visits. Data will be entered by the trained family members, clinicians and study personnel. eCRF data entry fields will be created that only allow data to be entered in a specific format (e.g., date fields and number fields). Automated checks implanted in the eCRF will check the data for plausibility and completeness while it is being saved. REDCap is a browser‐based software that will give continuous feedback to the person entering the data (e.g., erroneous or missing data). All data will be subject to monitoring by an independent reviewer.

### 3.11 Statistical analyses

The data will be analysed on an ‘intention to treat’ basis and analysed per protocol to verify the intention to treat results, as in previous similar studies (Aegerter et al., 2020). Missing data will be examined using the R‐package naniar 0.5 (Tierney, 2021) and multiple imputations will be used if required.

Linear mixed‐effects will be used to assess intervention effects on our primary outcome measured using the QUALIDEM over the study period. The broader category of generalized linear mixed‐effects models (GLMMs) will also be used to analyse secondary outcomes. GLMMs are extremely flexible since they support various distributions of the outcome variable, such as Gaussian, Bernoulli, binomial, gamma, negative binomial, ordinal and Poisson, as well as several link functions (e.g., identity, log, logit, probit and log‐log) (Cnaan et al., 1997; Lindstrom & Bates, 1990). Moreover, these models will handle non‐normal data and adjust the effects of clustering. Individuals will be nested in each cluster, and individuals will be measured at baseline and at five additional time points (Hemming et al., 2018; Luke, 2019).

Consequently, we will use GLMMs to investigate secondary outcomes, such as the symptoms and concerns of PWD. All models will be adjusted for potential confounding factors, such as age, gender, marital status, care dependence, dementia type and severity at baseline. All statistical analyses will be performed using R statistical software (R Core Team, 2020).

An alpha level of 0.05 will be accepted as significant. The results of the mixed‐effects modelling will be presented in outcome‐specific effect sizes, including the corresponding 95% confidence intervals.

### 3.12 Ethical considerations

This trial was registered at the German clinical trials register (DRKS00022339). The full registration can be accessed online at http://www.drks.de/DRKS00022339. The Zürich cantonal ethics board, as the lead board, reviewed the protocol, consent forms, educational material and eCRF (BASEC‐ID: 2019‐01847). The trial's scientific content and compliance with applicable research regulations (European Medicines Agency et al., 2016; World Medical Association, 2013) was confirmed. The ethics board in Zürich will coordinate with other cantonal boards in this multicentre study. The sponsor, the ethics committee or an independent trial monitor may visit the clinical and research sites for quality assurance. All involved parties are required to keep all participant data strictly confidential. In the event of an audit by a local or cantonal ethics committee, all source data, eCRFs and raw data will be made available to the auditors by the study team.

All raw data will be handled with the utmost discretion and will remain accessible to authorized personnel who require the data to fulfil their duties within the scope of the study only. On all documents and eCRFs, participants will only be identified by unique participant numbers. Source data will be kept at the clinical sites and will remain within the nursing homes. During a monitoring visit, eCRFs on an encrypted memory stick will be handed to the corresponding clinical site where the monitoring is to be conducted.

## 4 DISCUSSION

Currently, frontline staff in Swiss nursing homes need help for timely screening and reporting of needs and concerns observed in PWD (de Wolf‐Linder et al., 2018). When caring for PWD, they may observe complex and interrelated symptoms. Frontline staff needs to interpret, address and manage observed needs and concerns with an interprofessional team. Therefore, frontline staff need time and support to reflect and plan interactions, care or symptom management with other people, preferably also oriented to the person with dementia (Goodman et al., 2016). Furthermore, family members can provide valuable input and context for frontline staff to caring for the PWD and vice versa (Backhaus et al., 2020; Ellis‐Smith et al., 2018; Robertson et al., 2019).

In reviewing the literature, no research describing the symptoms and concerns and its management, be it palliative or curative, of PWD living in Swiss nursing homes is reported (Federal Council & Federal Office for Public Health, 2020). The Swiss federal office for public health (FOPH) and Swiss Alzheimer Society (2020) promote models for family member involvement for nursing homes, although frequency and form of exchange and participation are not determined and heavily based on family information provision members. FOPH made no specific recommendation for assessment and screening instruments for frontline staff other than the routine instruments. The relevant German guideline (Deuschl & Maier, 2016) refers to the nurse observation scale for geriatric patients (NOSGER) (Brunner & Spiegel, 2002), a scale for nurses to observe and rate behaviour that challenges, social behaviour, mood and functional impairment. However, neither NOSGER nor any of the routine assessment instruments reflect the person‐centred approach integral to palliative care, person‐centred care and caring quality in PWD (Lynette Chenoweth et al., 2019; Etkind et al., 2015; Howell et al., 2013). With IPOS‐Dem, a promising model for multi‐dimensional needs and concern assessment, improved communication, and improved caring quality, quality of care and quality of life in PWD has been described and identified (Ellis‐Smith et al., 2018).

Therefore, we propose to evaluate a novel case studies intervention based on accessible systematic observation of needs and concerns by family members and frontline staff. The case studies intervention is conducted by an expert nurse external to the nursing home, trained to encourage open discussion and bottom–up management needs and concerns in PWD participating in the study. We expect to encounter heterogeneity in Swiss nursing homes regarding the needs and concerns of PWD and organization, leadership, staffing and grade mix. Therefore, to account for the complexity of the intervention, we planned the study with an SW‐CRT design. SW‐CRTs have been shown to work well in the exploratory phase of developing complex interventions (Craig et al., 2008).

On the one hand, our decision to conduct an SW‐CRT reconciles the practical ethical and logistical concerns another trial may have posed for the PWD, us and the nursing home managers involved and the research team. On the other hand, there are drawbacks to employing this design: an SW‐CRT usually requires a larger sample size than the corresponding designs; also with the staggered starting times, the study duration is increased (Haines & Hemming, 2018; Hemming, Haines, et al., 2015). However, since blinding frontline staff and family members (i.e., the outcome assessors) is not possible in our study, we proceed with utmost care to reduce the risk of bias (Hemming et al., 2018; Siebenhofer et al., 2018). The increased sample size and, therefore, nursing homes contribute data on both intervention and control condition. The resulting datasets will be made accessible to interpret and apply to international long‐term care contexts.

## 5 CONCLUSION

This SW‐CRT will evaluate the effect of frontline healthcare staff and family members performing repeated structured assessment and subsequent case studies on the caring quality, symptoms and needs of PWD. Implementing the IPOS‐Dem may further encourage routine usage of outcome measures and foster more excellent staff–family communication. The use of the IPOS‐Dem will empower frontline staff in nursing homes and family members to report their insights about PWD. Furthermore, case studies led by specialist nurses and based on IPOS‐Dem‐informed professional and family input may improve quality of life and ameliorate the needs and concerns of PWD.

## 6 DATA AVAILABILTY STATEMNT

After Embargo for publications, the anonymized dataset will be made available on https://doi.org/10.5281/zenodo.4008427. We provide the statistical code under https://doi.org/10.5281/zenodo.4008429.

## CONFLICT OF INTEREST

The authors of the intervention trial declare no conflict of interest.

### PEER REVIEW

The peer review history for this article is available at https://publons.com/publon/10.1111/jan.14953.

## Supporting information

Fig S1

Fig S2
