# Supplementary figures and images for: Improving caring quality for people with dementia in nursing homes using IPOS‐Dem: A stepped‐wedge cluster randomized controlled trial protocol
Source: J Adv Nurs. 2021 Jul 7;77(10):4234–45. doi: 10.1111/jan.14953 (PMC8518061; doi:10.1111/jan.14953)

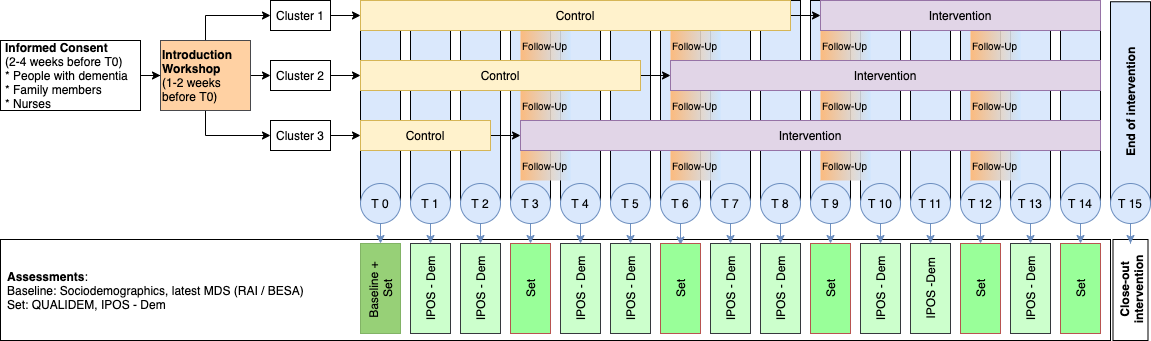


**Figure: Interventions and assessments in the overall context.**

Supplement: Supplementary file 1 — Fig S1 [file JAN-77-4234-s001.docx]

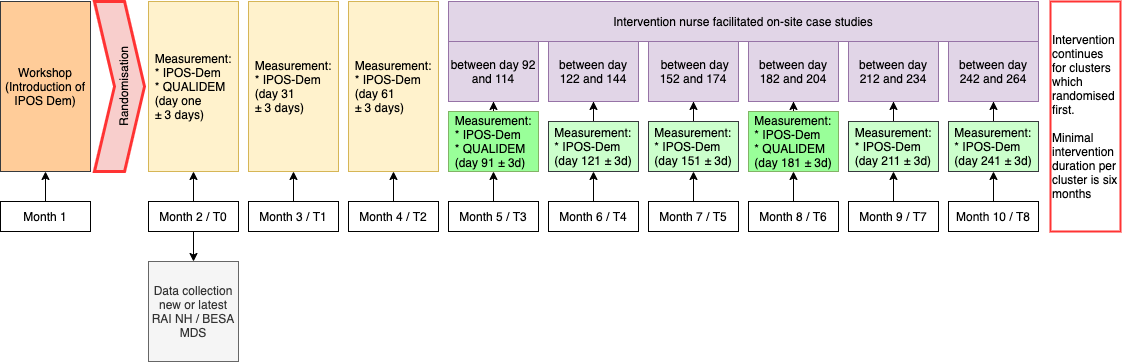


**Figure: Interventions and assessments per nursing home.**

Supplement: Supplementary file 2 — Fig S2 [file JAN-77-4234-s002.docx]
